# Borderline Personality Disorder: Bipolarity, Mood Stabilizers and Atypical Antipsychotics in Treatment

**DOI:** 10.4021/jocmr1042w

**Published:** 2012-09-12

**Authors:** Hasan Belli, Cenk Ural, Mahir Akbudak

**Affiliations:** a Psychiatry Clinic, Bagcilar Training and Research Hospital, Istanbul, Turkey

**Keywords:** Borderline personality disorder, Bipolar disorder, Mood stabilizers, Atypical antipsychotics

## Abstract

In this article, it is aimed to review the efficacies of mood stabilizers and atypical antipsychotics, which are used commonly in psychopharmacological treatments of bipolar and borderline personality disorders. In this context, common phenomenology between borderline personality and bipolar disorders and differential features of clinical diagnosis will be reviewed in line with the literature. Both disorders can demonstrate common features in the diagnostic aspect, and can overlap phenomenologically. Concomitance rate of both disorders is quite high. In order to differentiate these two disorders from each other, quality of mood fluctuations, impulsivity types and linear progression of disorders should be carefully considered. There are various studies in mood stabilizer use, like lithium, carbamazepine, oxcarbazepine, sodium valproate and lamotrigine, in the treatment of borderline personality disorder. Moreover, there are also studies, which have revealed efficacies of risperidone, olanzapine and quetiapine as atypical antipsychotics. It is not easy to differentiate borderline personality disorder from the bipolar disorders. An intensively careful evaluation should be performed. This differentiation may be helpful also for the treatment. There are many studies about efficacy of valproate and lamotrigine in treatment of borderline personality disorder. However, findings related to other mood stabilizers are inadequate. Olanzapine and quetiapine are reported to be more effective among atypical antipsychotics. No drug is approved for the treatment of borderline personality disorder by the entitled authorities, yet. Psychotherapeutic approaches have preserved their significant places in treatment of borderline personality disorder. Moreover, symptom based approach is recommended in use of mood stabilizers and atypical antipsychotics.

## Introduction

Although borderline statement has been used since the end of 1930s, it has been defined in 1980 as the second axis disorder category of published DSM-III (Diagnostic and Statistical Manuel of Mental Disorders, third edition) [1]. Gunderson has differentiated the diagnosis of Borderline Personality Disorder (BPD), which is defined by DSM and is believed to be present in the layer between neurotic and psychotic disorders, from borderline personality organization concept defined by Kernberg [1]. Kernberg analyzed theoretically the intrapsychic structure in his definition. He described a borderline personality disorder, which did not lose reality perception, displayed immature defenses and identity diffusion, and contained partially class A and partially class B personality disorders [2]. In DSM-IV, patients with symptoms covering from mood disorder spectrum to impulsivity spectrum have been defined. BPD is defined as a continuous pattern, which starts at the early adulthood and appears under various conditions; demonstrates inconsistencies in interpersonal relationships, self-perception and moods that are accompanied with prominent impulsivity [3]. Continuous struggling to abstain from being deserted, inconsistency in interpersonal relationships and self-perception, impulsivity, repetitive behaviors related to suicide, mood fluctuations, continuous feeling of emptiness, extensive anger, paranoid thought content, and sometimes severe dissociative signs may be observed characteristically in BPD [4].

There is an intensive effort to include BPD diagnosis into 1st axis disorders, because it has held a place in DSM classification. While some authors have tried to include BPD into a place in the schizophrenia spectrum [5], more researchers tried to relate it to mood disorders. Previously the disorder has been related to major depressive disorder [6], then strikingly attention is spent more on bipolar disorders, and this personality disorder is tried to be included into the bipolar disorder spectrum [7, 8].

Some researchers believed that bipolar disorder concept is used in a very narrow meaning; they have proposed that this diagnostic category, in fact, covers a larger spectrum [9, 10]. These researchers also proposed that many cases diagnosed with BPD belonged actually to the spectrum of mood disorders, and their treatments can be performed more efficiently if they are correctly diagnosed [11]. Contributions of Akiskal to this discussion were more concrete and descriptive. According to Akiskal, patients with BPD were actually people with cyclothymic clinical presentations, who continuously fluctuated between depression and irritable hypomania, which were darker or less stable state of bipolar II disorder. Therefore, they should be included into the bipolar spectrum and their treatments should be planned accordingly. Akiskal proposed that histrionic, narcissistic and borderline personality disorders accompanied by depression should be included into “Undistinguished or mild bipolar disorders” (Soft bipolar disorders) classification. Akiskal proposed that sometimes these disorders might progress from calmer presentations to more severe (Type 1 and Type 2) forms of bipolar disorders. Akiskal has tried to define bipolar spectrum in a very large diagnostic range. According to this theory, bipolar spectrum does not only cover mania or hypomania, but also emerging cyclothymic and hyperthymic temperaments. Both of these subtypes are featured under the “undistinguished or mild bipolar disorder spectrum”. Each phase is defined by a euthymic period lasting couple of days in cyclothymic or hyperthymic temperaments, which are started at the adolescent period or in early adulthood, and are defined with rapid cycling. This condition is designated as “ultra rapid cycling”. This designation is also a typical feature of mood fluctuations observed in borderline personality disorders. Therefore, Akiskal proposed that these cases should be evaluated in the bipolar spectrum [9].

Other authors did not accept these proposals. They stated that these disorders might be observed together, but BPD was a separate diagnostic category and DSM classification should be valid [12].

Various psychopharmacological agents have been tried against encountered symptoms of patients with BPD. Mood stabilizers and atypical antipsychotics have an important place among them. These drugs have been tried mainly on symptoms of impulsivity, anger, affective disorder, aggression, anxiety and depression. These drugs have been reported to be effective in some studies. However, their efficacies have been reported to be debatable in some other studies [13].

Both BPD and bipolar disorder are significantly severe and have significant potentials to affect various aspects of life. Both disorders can demonstrate common features for diagnosis and may overlap phenotypically. Quality of mood episodes, impulsivity types and linear progression of disorders should be carefully considered for the differential diagnosis. Correct diagnosis and planning the treatment accordingly are very important. Concomitant presence of these two conditions is not an uncommon condition and should not be missed.

According to the results of retrospective sign, it has been evident that concomitance of bipolar disorder and BPD does not affect clinical progression related to functional condition of BPD, remission levels and hospitalization rates. Concomitance of bipolar disorder and BPD is very significant for mood stabilizer use in the treatment, and may increase self-harming behaviors [14].

Mood stabilizers have been approved at least one of 3 phases of the bipolar disorder (mania, bipolar depression, long term maintenance) from FDA (Food and Drug Administration). However, no drug, including mood stabilizers and atypical antipsychotics, has been approved in treatment of BPD by FDA. Mood stabilizers and atypical antipsychotics have been recommended for use only for some symptoms [8]. This condition indicates that BPD being in the bipolar disorder spectrum does not have very much importance in the treatment.

Despite inadequate data in treatment of BPD with mood stabilizers and atypical antipsychotics, psychotherapeutic approaches, which may be evaluated as psychosocial treatments, are very promising. Dialectic behavioral therapy [15], schema focused therapy [16] and transference focused psychotherapies [17] can be mentioned among these approaches. As it is observed, efforts trying to place BPD in between bipolar disorders do not provide any significant contributions for treatment approaches. Significant overlapping of diagnostic criteria in DSM system does not imply that both of these disorder groups are exactly the same. However, it is a fact that bipolar disorders especially other than bipolar I disorder are not easily differentiated from BPD. Undetailed differentiation efforts, which are purely based on diagnostic criteria of DSM system, were inadequate. Differentiation with a more comprehensive evaluation might also be beneficial for the treatment.

In this present review article, it is aimed to discuss overlapping phenomenology of BPD and bipolar disorder and differential clinical diagnosis. Additionally efficacies of mood stabilizers and atypical antipsychotics used in psychopharmacological treatment of BPD will also be discussed.

## Borderline Personality Disorder and Prevalence of Bipolar Disorders

According to DSM-IV-TR, prevalence rate of BPD in the general population is estimated to be around 2%. This rate is about 1-2% for bipolar disorder. Other estimations give this rate as 5% for bipolar spectrum disorders [17].

In the review studies, which have been compiled comprehensively by Paris et al, it has been emphasized that bipolar I disorder rate among BPD patients was 5.6-16.1%. This rate was nearly 9.2%. The rate is defined 8-19% when bipolar II disorder is considered. The mean rate was 10.7% [18]. When 2 studies with adequate sample sizes, which had 6 years’ linear follow up phases, and were performed by structured diagnostic interviews of strong methods, have been reviewed, new-onset bipolar disorder has been detected in patients with BPD at low rates. However, these rates were not different from those in the control group [6, 19]. When another methodologically well-designed study has been reviewed, bipolar I and II disorders have been detected at significantly higher levels in patients with BPD than groups composed of other personality disorders (schizotypal, avoidant and obsessive-compulsive personality disorders). These rates were 19.4% for BPD and 7.9% for the other group. When the groups have been linearly followed up for 4 years, initiation rates of bipolar I and II disorders in BPD group were significantly higher than the rates in the other personality disorders group. The rates were 8.2% and 3.1%, respectively. Although these rates have demonstrated a moderate level of risk in patients diagnosed with BPD, this rate was quite lower than that of in major depressive disorder and substance abuse, which are detected in BPD [14].

Authors investigating BPD rate in bipolar I disorder have reported very different results, which differed in the range of 0.5% to 30% with a mean value of 10.7%. However, these rates in bipolar II disorder are differed in the range of 12-23%, and the mean value was 16.6%. In a study investigating the relationship between cyclothymia and BPD, concomitance rate of both disorders has been defined quite high. This rate was 62% [20].

## Diagnosis and Differential Diagnosis

It is quite difficult to differentiate between bipolar disorder and BPD, because both disorders progress with affective disorder, irritability and impulsivity. When DSM-IV-TR criteria are compared with themselves, it is obvious that there is a prominent overlap. Phenomenology of mania is prominently different from BPD. Prominent psychic and motor accelerations, psychosis and irritability can be differentiated in factor analysis of manic symptoms [21, 22]. When factor analyses of BPD are performed, 3 factors are detected characteristically; deterioration of interpersonal relationships, behavioral disorder or inconsistency and affective disorder [23, 24].

In recent studies, a couple of parameters have been defined to differentiate the overlap between these two disorders. These parameters are described as quality of mood episodes, type of impulsivity and linear progression of disorders.

### Mood episodes

Both disorders cause mood fluctuations and affective mobility, but phenomenology of mood episodes is different. Fluctuant mood and negative affect are characterized in BPD. This condition can be triggered by perceived stress or stress factors originating from interpersonal relationships. It is transient, mainly dependent on environmental factors in the nature and can last from a couple of minutes to hours. Mood fluctuations in bipolar disorder is longer and much more spontaneously. There are much more elongated excitement periods especially in bipolar disorder type I. In addition to this, affective variability is the characteristic aspect of emotional response. According to obtained data, affective problems last for life-long; they are characteristic in childhood and even in the babyhood. However, there are also asymptomatic intervals in bipolar disorder [25, 26].

Mood fluctuations in BPD and bipolar II disorder are also differentiated by types of emotions. People diagnosed with BPD have demonstrated fluctuations from euthymia to anger, where euthymia was not frequent. However in bipolar II, affective shift was from euthymia to excitement or rising. Shift in BPD is triggered generally by interpersonal stress factors that are characterized by rejection or desertion. These conditions are quite seldom in all borderline disorders [27]. Differentiation between BPD and rapid cycling bipolar disorder is still a problem. While durations of mood episodes, qualitative emotional shift, repetitive triggering findings and detailed linear pattern evaluation help the differential diagnosis between BPD and bipolar disorders; this condition does not prevent from continuous severe difficulties in diagnosing rapidly cycling bipolar disorder forms [27-29].

### Impulsivity

Impulsivity is observed both in BPD and bipolar disorder. The differential feature of impulsivity is unplanned motor impulsivity in the manic phase, whereas in the depressive phase it is characterized again by intense and unplanned impulsivity. The impulsivity is also dominantly unplanned in character in BPD. Data have indicated that existent symptoms were mainly overlapping with depressive pole of the bipolar disorder [30-32].

Likewise, BPD can be differentiated from bipolar II by the presence of hostile thoughts and difference in impulsivity. In bipolar II, impulsivity is related to the attention. This impulsivity can easily be switched to another direction and be away from a target. There is an unplanned impulsivity in BPD. The highest impulsivity rates in the population are detected in people, who have concomitant BPD and bipolar II disorders. It has been claimed that these subjects have the highest levels of self-harming behaviors. These findings have indicated that both disorders can be diagnosed together under appropriate circumstances [32]. Clinically, impulsivity in BPD is believed to have a more episodic character than the impulsivity in bipolar disorder. However, some conditions, like substance abuse, can cause bipolar disorder to become more complicated, and impulsivity can be observed between the episodes in these concomitant cases [33]. Impulsive actions, like suicide commitment, can be observed in both disorders, but it is generally more common in the depressive phase of bipolar disorder. It is generally related to hopelessness in BPD and it is frequently resulted from incompetence of coping with stressed situations [34-36].

### Linear progression of disorders

When linear progression of disorders, based on axis I and axis II, are compared traditionally; mood disorders have been accepted to be cyclic and treatable in nature, whereas personality disorders are sustained for life-long and are resistant to treatment. However, it has been reported in many studies that bipolar cases could also have a chronic progression, demonstrate long term disease signs, and symptoms could become chronic prominently between the episodes. Moreover, results of follow up studies with longer durations have revealed that majority of subjects diagnosed with BPD have not met the disorder criteria after many years [37, 38]. However, core signs existent below the threshold level have continued in BPD. Although more dramatic and harmful behaviors are subsided, psychopathological signs in affective and interpersonal relationship areas were permanent. However, cases which have not got into even partial remission, may also be observed [39, 40].

## Use of Mood Stabilizers in Treatment of Borderline Personality Disorder

Psychotherapy preserves its significance at the center of BPD treatment. However, pharmacotherapy can be recommended in some situations [41, 42]. There is information that some medications are effective in some symptom clusters and crises. Mood stabilizers have an important place among these medications [43].

### Mood stabilizers

#### Lithium carbonate

Lithium is compared with desipramine in 10 patients diagnosed with BPD in a 6-week double blind, placebo controlled study. In this study, it has been reported that lithium was effective against basic psychopathological features of BPD; irritation, anger and self-harming behavior [44]. In a review study designed by Stein, lithium and carbamazepine have been effective on behavioral deterioration and aggressiveness observed in patients with BPD or antisocial personality disorder [45].

#### Carbamazepine

Gardner and Crowdry have demonstrated in their study with 11 female patients that carbamazepine has decreased frequency and severity of uncontrolled behaviors [46]. These results have been confirmed by other studies. One of them has been provided in the 6-week, double blind, controlled study in which carbamazepine (820 mg/d) has been compared with alprazolam (4.7 mg/d), trifluoperazine (7.8 mg/d) and tranylcypromine (40 mg/d). In this study, 16 patients with hysteroid dysphoria with BPD have been included and efficacy of carbamazepine has been confirmed [47].

Controlled trials have demonstrated that carbamazepine has helped not only the impulsive aggressiveness, but also the affective fluctuations [48, 49]. Denicoff et al performed a study on 1,257 subjects with various neurological and psychiatric disorders, and compared carbamazepine, lithium, valproate, clonazepam, calcium channel blockers, phenytoin, antipsychotics and electroconvulsive therapy. In this study, carbamazepine has been demonstrated to have a significant superiority in total recovery scores with BPD diagnosis subgroup than the others [50].

#### Oxcarbazepine

In a 12-week pilot study, 13 subjects from outpatient clinic with BPD were enrolled into the study. Patients received oxcarbazepine and statistically significant improvements have been detected in signs of anxiety, interpersonal relationships, impulsivity, affective fluctuations and anger [51].

#### Divalproex sodium and valproate

Divalproex sodium is among the mood stabilizers, which are comprehensively studied in patients with BPD [52]. Wilcox claimed that divalproex decreased agitation significantly in patients with BPD. He detected prominent decreases in agitation signs in patient groups with BPD and bipolar disorder [53]. These findings have been confirmed by studies, which have been performed later with the use of divalproex sodium [54].

Then 3 placebo controlled studies have been performed. In a double blind study performed by Hollander et al, valproate has been used at the plasma level of 80 g/mL in 16 patients with BPD, despite prominent improvements in global symptomatology, prominent decreases have been reported in depressive symptoms, aggression, irritability and suicidal idea or behaviors [55]. In a sequential double blind study of 12 weeks duration, into which 52 patients attending the outpatient clinic are enrolled, efficacy of valproate (mean daily dose of 1,325 mg) is confirmed on impulsive aggression [56]. In the 6-month controlled study of Frankenburg and Zanarin, which contained 30 BPD patients concomitantly diagnosed with bipolar II, prominent effects of valproate (plasma levels were in 50 - 100 g/mL) have been detected on interpersonal sensitivity, anger, hostility and aggression [57].

#### Lamotrigine

Lamotrigine use in treatment of BPD has started by a study from Pinto and Akiskal. Open ended study contained 8 patients, who are followed up for one year and at the outpatient clinic. Significant improvements in total functions were around 40% in the study. Moreover, prominent improvements have been reported in sexual impulsivity, substance abuse and suicidal behaviors [58]. In a review article prepared by Green, it has been reported that this drug, which was effective in mood disorders, was also effective in balancing the moods in patients diagnosed with BPD [59].

Preston et al detected that around 40% of 35 bipolar patients was diagnosed with BPD, when they were evaluated for concomitant BPD diagnosis retrospectively. These patients have been evaluated for lamotrigine efficacy in two open ended studies. Results have demonstrated that lamotrigine was effective in all characteristic features of BPD and especially improved impulsivity and mood fluctuations prominently [60].

In a more recent study, Tritt et al enrolled 24 female patients, who met BPD criteria and compared lamotrigine and placebo. After 8 weeks, they detected prominent improvements in anger control in patients treated with lamotrigine [61].

#### Atypical antipsychotics

Atypical antipsychotics block both dopamine and serotonin (5-HT2) receptors. These drugs demonstrate antimanic effects by dopamine receptor blockage, and anti-depressant effects by 5-HT2 antagonism. Therefore, atypical antipsychotics have been proposed to be effective in the treatment and prevention of bipolar mania and depression [62]. These hypotheses have been confirmed by a series of studies [63].

Clozapine has been found to be effective in all symptoms, especially aggression and severe psychotic symptoms related to BPD [64-68]. However, the patients, who were treated with clozapine, had frequently Axis-I disorder comorbidity and were resistant to the previous treatments.

Risperidone related studies have been more frequently obtained from case presentations and one open labeled study [69-72]. Rocca et al reported from their 8-week open label study, into which they enrolled 15 patients with BPD that risperidone (mean dose 3.3 mg/d) was effective in aggressive behaviors, affective fluctuations and global psychopathology [72].

Schulz et al investigated efficacy of olanzapine in an open label study with 9 patients with dysthymia and comorbid BPD diagnosis. Prominent improvements have been reported 8 weeks after the treatment in impulsivity, hostility, global psychopathology and global functions [73]. After that, a couple of controlled studies have been published in olanzapine efficacy. Zanarini and Frankenburg demonstrated in their 6-month double blind, placebo controlled study on 28 female patients with BPD that olanzapine (mean dose 5.33 mg/d) was effective in anxiety, paranoid thoughts and interpersonal sensitivity [74]. Bogenschutz and Nurnberg tried olanzapine (doses ranging 5 - 10 mg/d) in 40 patients with BPD in another 12-week, double blind, placebo controlled study. They detected prominent improvements in borderline psychopathology and ager in the patients [75]. Zanarini et al compared fluoxetine, olanzapine and olanzapine-fluoxetine combinations in 45 female borderline patients. This study was designed as 8 weeks, double blind study. Investigators detected that all three treatments improved prominently chronic dysphoria and impulsive aggression. Moreover, they reported that olanzapine monotherapy and olanzapine-fluoxetine combination were significantly superior to fluoxetine monotherapy [76]. Soler et al compared olanzapine and placebo in combination with dialectic behavioral therapy. This 12- week study was composed of 60 subjects, who were attending the outpatient clinic. Investigators reported that olanzapine (mean dose 8.8 mg/d) caused prominent decreases in impulsive aggressive behavior, depression and anxiety [77].

Another atypical antipsychotic, which has been investigated in BPD treatment, is quetiapine. Schulz evaluated quetiapine (between 25 and 300 mg/d) in an open label study of 8 weeks’ duration with 10 patients. Results have indicated that quetiapine caused prominent improvements in hostility, impulsivity and social functions [78]. Villeneuve and Lemellin reported similar findings in their studies. They studied quetiapine (doses of 175 and 400 mg/d) in a 12-week study on 23 patients, who have been followed up at the outpatient clinic. These investigators reported that quetiapine had significant improvements in impulsivity, hostility, depression, anxiety and social functions [79]. Bellino et al conducted a 12-week pilot study on 14 patients, and they investigated treatment efficacy of quetiapine (mean dose 309 mg/d). Findings were consistent at a great extend with the previous studies. Investigators reported that quetiapine caused prominent improvements in global symptoms, impulsivity, anger explosions, anxiety and social functions [80].

## Conclusion

According to conducted studies, BPD is diagnosed 10% of outpatient clinic patients and 20% of hospitalized ones. BPD is accompanied at significant levels by psychosocial deteriorations and mortality related suicide attempts. More than 10% of patients have attempted suicide. This rate is approximately 50 folds increased than in the general population [81]. These rates have indicated the significance of efficient therapy for BPD.

Bipolar disorder and BPD are closely related psychological disorders both in phenomenology and treatment responses. There are difficulties to define similar and differential features of them. However, if the common symptomatology is considered, despite the central place of psychotherapeutic interventions in the treatment, there are areas in BPD treatment that mood stabilizers and atypical antipsychotics may be helpful. According to the literature, there are more data related to efficacies of valproate, lamotrigine, olanzapine and quetiapine. There are quite a few data related to other drugs. In this context, further placebo controlled, blind studies, which will introduce separate official approval procedures for each mood stabilizer and atypical antipsychotics, are required.
